# Stage-Dependent Embryolethality of Diclofenac Sodium: Quantitative Assessment of Dose–Time Interaction and Critical Windows of Susceptibility in the In Ovo Chicken Embryo Model

**DOI:** 10.3390/vetsci13050492

**Published:** 2026-05-19

**Authors:** Harun Kizilay, Seyma Tetik Rama

**Affiliations:** Department of Pharmacology, Faculty of Pharmacy, Selcuk University, Konya 42130, Türkiye; seyma.tetik@selcuk.edu.tr

**Keywords:** diclofenac sodium, critical windows of susceptibility, potency ratio, chicken embryo, in ovo, LD50

## Abstract

(1) Background: Diclofenac sodium is a drug with reported developmental toxicity in several non-mammalian and mammalian models. This study aims to evaluate the stage-dependent embryolethality of diclofenac sodium according to developmental stage using the chicken (*Gallus gallus domesticus*) embryo in ovo model, a system widely used in veterinary and avian developmental toxicology screening. The study focuses specifically on quantitatively determining the “critical sensitivity windows” between the early (day 7) and late (day 14) embryonic stages. (2) Methods: Fertilized chicken eggs (*Gallus gallus domesticus*) were exposed to different doses (3.125–50 mg/kg) of diclofenac sodium, and mortality rates were analyzed. (3) Results: The data indicated that diclofenac toxicity is highly dependent on the developmental stage (*p* = 0.002). While the LD50 value for the early stage (day 7) was calculated as 20.67 mg/kg, (95% CI 6.79–860.87 mg/kg; wide interval reflecting low precision and steep response), embryos at the late stage (day 14) were found to be more resistant, with an LD50 value of 32.16 mg/kg (95% CI 27.77–37.90 mg/kg). The calculated Potency Ratio of 1.55 indicates that 7-day-old embryos are more sensitive to diclofenac. (4) Conclusions: This study provides model-specific preliminary quantitative data on the stage-dependent embryo mortality profile of diclofenac sodium in chicken embryos. The higher mortality observed on day 7 is consistent with a window of susceptibility during early organogenesis that may be associated with immature detoxification pathways (e.g., hepatic CYP450) and active organic anion transport (e.g., Oatp1d1) reported in other developmental models. However, these mechanisms were not directly measured here. These findings contribute to comparative developmental toxicology and the One Health-focused assessment of NSAID exposure in animal species, emphasizing the importance of considering “timing of exposure” in developmental toxicity assessments. Furthermore, no extrapolation to mammalian pregnancy or human clinical guidelines is implied.

## Simple Summary

Diclofenac sodium, a nonsteroidal anti-inflammatory drug (NSAID), is among the most widely used medications worldwide. However, the safety of the effects of NSAIDs during avian and mammalian embryonic development has not yet been fully elucidated; this has significant implications for the evaluation of veterinary developmental toxicology and drug exposure within the “One Health” framework. In this study, the effect of the timing of diclofenac exposure on the embryonic period was investigated using in ovo methods with fertilized chicken eggs. We exposed the eggs to different doses of diclofenac at two critical developmental stages—day 7 (organogenesis) and day 14 (late development)—and measured mortality rates. It was found that embryos during the early organogenesis stage were 1.55 times more sensitive to diclofenac than embryos in the late stage. Within the limitations of this in ovo chicken embryo model, the findings indicate that the early organogenesis stage is significantly more sensitive to diclofenac-induced embryo death compared to the late embryonic stage. The results provide model-specific preliminary data for comparative developmental toxicology, supporting One Health-focused risk assessments of NSAIDs in animal species and potentially informing veterinary drug safety screening. However, specific mammalian and clinical studies are required to make direct generalizations to mammalian pregnancy or clinical prescribing.

## 1. Introduction

Diclofenac sodium is a nonsteroidal anti-inflammatory drug with analgesic, anti-inflammatory, and antipyretic effects [1]. The mechanism of action is to suppress prostaglandin synthesis through the inhibition of cyclooxygenase (COX) enzymes. The pharmacological effects of NSAIDs, which suppress prostaglandin synthesis, their primary mechanism of action, were first elucidated by Vane [2].

Diclofenac is frequently used in clinical practice for the treatment of chronic inflammatory diseases such as rheumatoid arthritis, osteoarthritis, ankylosing spondylitis, traumatic musculoskeletal injuries, and postoperative pain [3]. Diclofenac, available in oral, parenteral, topical, and ophthalmic forms, exhibits pharmacokinetic properties that vary depending on the route of administration. Diclofenac has higher selectivity for the COX-2 enzyme compared to the COX-1 enzyme, and gastrointestinal and renal side effects may occur due to COX-1 enzyme inhibition [4]. Long-term or high-dose use of diclofenac causes a number of side effects. These primarily include gastrointestinal ulceration, gastric mucosal bleeding, and irregular inflammatory response [5]. The U.S. Food and Drug Administration (FDA) has issued serious warnings that NSAID use after the 20th week of pregnancy may cause fetal kidney dysfunction and oligohydramnios [6]. The adverse effect of diclofenac on renal function is primarily based on the suppression of prostaglandin synthesis, which is necessary to maintain renal hemodynamics and modulate afferent arteriolar tone. Sivaraj and Umarani [7] demonstrated that diclofenac-induced inhibition causes a significant decrease in glomerular filtration rate (GFR), while Maideen et al. [8] confirm that this mechanism leads to severe oligohydramnios and premature ductus arteriosus closure, particularly when exposure occurs after the 20th week of pregnancy. Additionally, a large-scale cohort study by Tain et al. [9] revealed that exposure during pregnancy is not only associated with impaired fetal nephrogenesis but also significantly linked to an increased risk of chronic kidney disease (CKD) in childhood, confirming previous case reports of irreversible functional impairment [10]. Additionally, a large-scale cohort study covering more than 151,000 pregnancies highlighted the adverse effects of over-the-counter analgesic use during pregnancy on perinatal health and indicated that the developmental safety profiles of these drugs need to be reevaluated [11].

Due to its widespread use, diclofenac has been identified as a “new generation pollutant” that accumulates in aquatic ecosystems and threatens the development processes of organisms at different levels of the food chain (zooplankton and phytoplankton) [12]. It is known that even at low concentrations, diclofenac causes behavioral disorders and physiological stress responses in aquatic organisms and produces neurotoxic effects [13]. Another study indicates that even the byproducts of diclofenac photolysis, which are abundant in wastewater, increase glutathione-S-transferase and catalase enzyme levels in adult zebrafish and cause oxidative stress [14]. The toxicological risks of diclofenac extend far beyond human health. The drug bioaccumulates, posing a serious threat to non-target species at various trophic levels. Indeed, the dramatic decline in vulture populations due to diclofenac-induced kidney failure has been identified as the most concrete and striking evidence of this ecological hazard [15].

Experimental studies conducted on whole rat embryo cultures indicate that diclofenac exposure significantly increases embryonic 8-isoprostaglandin F2α levels, which play a central role in the formation of oxidative damage resulting from an increase in free oxygen radicals that cause embryotoxicity [16]. Similarly, Ertekin et al. [17] demonstrated that exposure to sodium diclofenac disrupts neurulation in chicken embryos and leads to neural tube defects characterized by midline closure defects, as well as other developmental abnormalities such as a reduction in crown-rump length and a decrease in somite formation.

Although numerous studies have been conducted on the environmental and clinical toxicity of diclofenac, the effect of exposure duration during embryogenesis on mortality remains unclear. Although the embryotoxic effects of diclofenac have been demonstrated in different models in the literature, the differences between the “windows of susceptibility” during early organogenesis and fetal growth stages and the mortality thresholds (LD50) between these stages have not yet been fully defined.

The in ovo method is notable for being more practical and economical compared to traditional animal testing. This method provides reliable endpoints for assessing embryotoxicity and offers a suitable approach for the initial evaluation of potential teratogenic substances [18]. Additionally, toxicity studies conducted using the in ovo method remain an important method that is fully consistent with the 3Rs principles advocated by Russell and Burch [19] in animal experiments, specifically the “Replacement” principle. This study aims to determine the toxicity profile of diclofenac on the developing embryo, its critical risk windows, and time-dependent toxicity by conducting a comparative analysis using the in ovo model. The chicken embryo model in eggs is a well-established system in the fields of veterinary medicine and avian developmental toxicology and is recognized as an ethically sound alternative for screening drugs and environmental pollutants. In this context, this study contributes to comparative developmental toxicology and an evaluation of NSAID exposure in various animal species—including poultry and other bird species relevant to veterinary medicine—based on the “One Health” approach while also complementing existing findings in mammals and aquatic organisms.

## 2. Materials and Methods

### 2.1. Ethical Statement

This study was approved by the Selcuk University Animal Experiments Local Ethics Committee (Approval no: 2025/50).

This study was designed as a prospective, controlled, and dose-escalation in ovo experimental study. The experimental procedure was divided into two separate phases based on the timing of drug administration: day 7 and day 14. Embryonic development stages were determined according to the Hamburger and Hamilton [20] staging scale. Incubation periods were completed in an incubator (Imza Teknik, Konya, Türkiye) set at 55% humidity and 37.8 ± 0.2 °C.

To ensure proper development, the eggs were automatically rotated every 90 min at a 45-degree angle. Viability and fertilization status were assessed by ovoscopy. The replacement of non-viable or unfertilized eggs was performed only prior to drug administration. No further replacements were made after drug administration began. The replaced eggs were taken from the same incubation group, and to maintain the randomization design, all eggs were re-randomized into dose groups using a computer-generated random sequence. A commercial diclofenac sodium formulation (Lafenac^®^, Alke Health Products, Tokat, Türkiye; a colorless, sterile solution containing 50 mg/mL diclofenac sodium, excipients: sodium metabisulfite (E223), propylene glycol, benzyl alcohol (E1519), N-methyl-2-pyrrolidone, sodium hydroxide, water for injection) was used. A registered injectable formulation was used instead of a pure diclofenac sodium reference standard because it more accurately reflects the form encountered in veterinary and human exposure. The stock solution was serially diluted with sterile 0.9% NaCl to obtain target doses of 3.125, 6.25, 12.5, 25, and 50 mg per kilogram of egg weight (calculated based on individual egg weight measured using a calibrated digital scale prior (Ohaus CS 200, Beijing, China) to treatment. All doses were administered in a fixed volume of 50 μL. A vehicle (saline) control group receiving an equivalent volume of sterile 0.9% NaCl prepared with the same diluent was used as the most appropriate comparison group. Although the effects of the vehicle cannot be entirely disregarded, the absence of mortality in the saline control group supports that the response is primarily due to diclofenac. The dose range (3.125–50 mg/kg egg weight) was selected based on: (i) previous in ovo and zebrafish embryo studies reporting developmental effects within this concentration range [17,21]; (ii) the half-log interval logic enabling probit-based LD50 estimation; and (iii) preliminary range-setting observations conducted in our laboratory.

### 2.2. Animal Experiments

**Figure 1:**
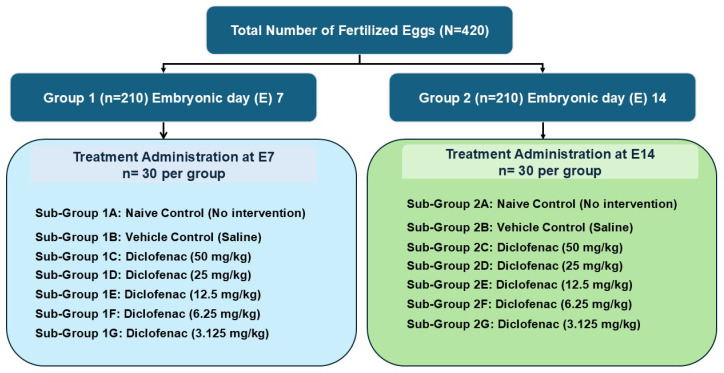
Experimental design of the study.

A total of 420 commercially available Babcock White hybrid chicken eggs (*Gallus gallus domesticus*) were used for the study, selected for their homogeneity and reproducibility of embryonic development, and which were specific pathogen free (SPF) and fertile. The eggs were randomly divided into two groups: group 1 (*n* = 210) treated on the 7th day of embryonic development and group 2 (*n* = 210) treated on the 14th day of embryonic development. The fertilized eggs in groups 1 and 2 were randomly divided into 7 equal groups (*n* = 30) and placed in an incubator. Diclofenac was administered to the groups on the 7th and 14th days of embryonic development (Figure 1).

**Figure 2:**
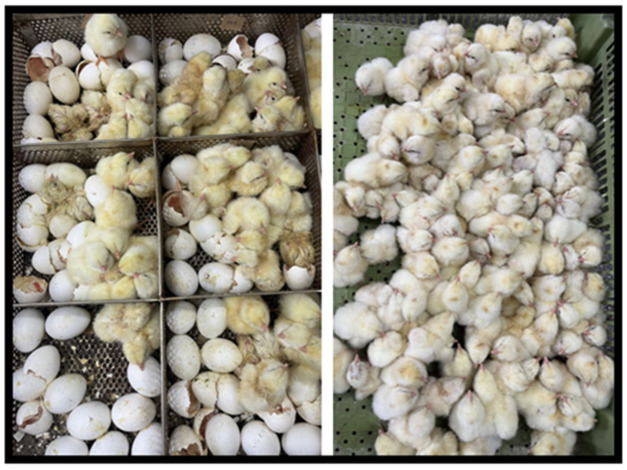
Evaluation of embryolethality in fertilized eggs administered with diclofenac.

To allow the applied drug to diffuse, no turning was performed for 1 h after application. All eggs were kept under optimal conditions in the incubator to ensure absorption of the drug through the air sac. Specifically, a diluted formulation (50 μL) was injected into the air sac of the egg. Prior to each injection, the shell surface was disinfected with 70% ethanol, and a small (~1 mm) opening was created at the center of the blunt end of the egg using a sterile needle. The diclofenac solution was then administered through this opening using a sterile 1 mL insulin syringe attached to a needle inserted to a depth of approximately 5 mm, ensuring that the solution was delivered into the air sac cavity without coming into contact with the chorioallantoic membrane, the yolk, or the albumen. Immediately after injection, the opening was sealed with sterile paraffin wax to prevent leakage and minimize water and gas exchange. All procedures were performed in a laminar flow cabinet using sterile gloves and instruments. The air sac route was selected because it allows for systemic exposure via diffusion and has been previously used in chicken embryo developmental toxicology studies, including those conducted in our laboratory [17,22]. At the end of the 21-day incubation period, the eggs were candled and embryotoxicity was assessed (Figure 2). To classify embryo deaths, additional light examinations were performed daily from the day of drug administration until the 21st day of the incubation period. Early embryo death (EED) was defined as embryo death occurring between days 0 and 7 of the incubation period (as indicated by the absence of circulation, vascular network, or movement during light examination), while late embryo death (LED) was defined as death occurring between days 8 and 21. At the end of the 21-day incubation period, all eggs were hatched and examined macroscopically to verify the classification based on light examination. Embryos that completed their development without detectable mortality by day 21 were recorded as the number of normal live embryos (NAEs) at the end of incubation.

### 2.3. Statistical Analysis

The actual mortality rate in the study was determined using the Abbott method on embryonic mortality rates [22,23]. Differences in mortality rates between experimental groups, and between Day 7 and Day 14 within the same dose group, were analyzed using the Pearson chi-square test (SPSS v22.0; IBM Corp., Armonk, NY, USA). When the expected cell count was less than 5, Fisher’s exact test was applied instead. The Holm–Bonferroni correction was used to control the familywise error rate across all pairwise comparisons by dose. For the logistic regression analysis of embryo mortality, the binary outcome (alive = 0, dead = 1) was the dependent variable. Dose (continuous, log10-transformed mg/kg egg weight) and treatment day (Day 7 vs. Day 14, coded as a binary indicator with Day 14 as the reference category) were included as independent variables, along with their two-way interaction term. Results were reported as odds ratios (ORs) with 95% confidence intervals (CIs). The embryolethal dose 50 (LD50) and its 95% CI were estimated separately for the Day 7 and Day 14 datasets using the probit method (SPSS v22.0). The Efficacy Ratio (LD50 Day 14/LD50 Day 7) was calculated as a measure of relative sensitivity between treatment days, and its 95% confidence limits were estimated using Fieller’s theorem. Sigmoidal dose–response curves were fitted using GraphPad Prism v8.0 (GraphPad Software, San Diego, CA, USA). All tests were two-sided, and a *p*-value of less than 0.05 was considered statistically significant. *p*-values were reported consistently throughout the article (e.g., *p* < 0.05, *p* = 0.002).

## 3. Results

The mortality rates following diclofenac administration on embryonic day 7 and embryonic day 14 are presented in Table 1.

On the 7th day of drug administration, the embryo mortality rate was determined to be 93.33% and the survival rate 6.67% in the group receiving a high dose of diclofenac (50 mg/kg). At a dose of 25 mg/kg, the mortality rate was 86.67% and the survival rate 13.33%. At a dose of 12.5 mg/kg, the mortality rate was 33.33%, and the survival rate was 66.67%. No deaths were observed in the experimental groups at doses of 6.25 mg/kg or 3.125 mg/kg (Table 1).

The results of the 7th day applications showed that the mortality difference between the control group and the high-dose groups (25 and 50 mg/kg) was statistically significant (*p* < 0.05). Logistic Regression analysis results revealed that the risk of embryo death was significantly affected independently of both dose and application day. The effect of the application day (day 7 vs. day 14) on survival was found to be statistically highly significant (OR: 0.42, *p* = 0.002).

As a result of the 14-day applications, the embryo mortality rate was determined to be 86.67% and the survival rate 13.33% in the group given high-dose diclofenac (50 mg/kg). At a dose of 25 mg/kg, the mortality rate was found to be 40.0% and the survival rate 60.0%. No mortality was observed at a dose of 12.5 mg/kg. Both at 6.25 mg/kg egg weight and 3.125 mg/kg doses, the mortality rate was 3.33% and the survival rate was 96.67% (Table 1). As a result of the 14-day applications, a dose-dependent increase in mortality (i.e., decrease in survival) was observed in the late period.

The Potency Ratio calculated to determine the sensitivity difference between two different developmental stages was found to be 1.55. This value indicates that 14-day-old embryos are 1.55 times more resistant to diclofenac toxicity compared to 7-day-old embryos. Given the wide LD50 confidence interval for the 7-day values, this estimate of relative sensitivity should be interpreted with caution and is best considered as supportive evidence rather than definitive proof of a stage-dependent effect.

According to the probit test performed, the LD50 dose of diclofenac administered on day 7 was found to be 20.67 mg/kg (95% CI 6.79–860.87 mg/kg). It should be noted that this confidence interval is quite wide and that the precision of the point estimate for the Day 7 LD50 is low. The most plausible explanation for this wide range may be the steep dose–response relationship and the absence of partial responses (i.e., doses yielding a mortality rate of 0% to ~33%, followed by ~87%) in the day 7 dataset, which limits the resolution of the probit fit. Therefore, the numerical precision of the 7-day LD50 value should be treated with caution, and the finding should be interpreted as a ‘guideline’ estimate. However, despite this uncertainty, the mortality differences on Days 7 and 14 (12.5 and 25 mg/kg, *p* < 0.001), as demonstrated by Fisher’s exact test, confirm a robust time-dependent effect that is unaffected by the loss of precision in the LD50 calculation. The LD50 dose of diclofenac administered on day 14 was determined to be 32.16 mg/kg (95% CI 27.77 to 37.90 mg/kg).

## 4. Discussion

**Figure 3:**
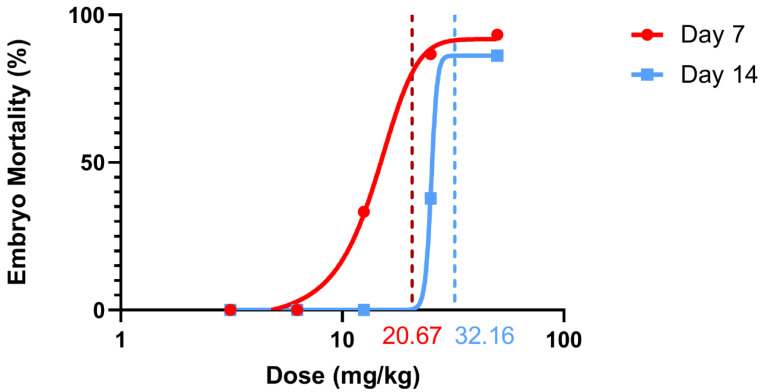
Sigmoidal dose–response curves of diclofenac sodium in early (Day 7) and late (Day 14) chicken embryos following in ovo administration. Mortality rates (Abbott-corrected) are plotted against log-transformed doses (mg/kg egg weight). The leftward shift of the Day 7 curve indicates markedly greater sensitivity during organogenesis. Vertical lines denote calculated LD50 values: 20.67 mg/kg (Day 7) and 32.16 mg/kg (Day 14). Curves were fitted using GraphPad Prism v8.0.

This study was conducted to evaluate the potential embryotoxic effects of diclofenac sodium administered at different doses to chicken embryos using the in ovo method. The data indicates that diclofenac toxicity is not a fixed event but a dynamic process related to the developmental period. Sigmoidal Dose–Response Curves are presented to describe this dynamic interaction (Figure 3). The leftward shift and steeper slope of the 7-day curve indicate a clear “critical sensitivity window” during early organogenesis. This increased sensitivity is quantitatively evidenced by the significant difference in LD50 values (20.67 mg/kg vs. 32.16 mg/kg) between the early organogenesis stage (day 7) and the late fetal period (day 14).

The high incidence of early-stage mortality observed in this study is consistent with the known sensitivity of the in ovo model to chemical exposure during critical developmental windows. This sensitivity has also been confirmed by Canbar, Akcakavak, Uslu, Arslan and Kizilay [22], who recently demonstrated that developmental exposure to environmental toxins such as cadmium can cause serious teratogenic effects, including microphthalmia and beak deformities. This also confirms that it is a reliable indicator for detecting specific embryotoxicity during the developmental stage of chick embryos. A similar toxicological course has recently been demonstrated in the freshwater shrimp *Macrobrachium borellii*. In a study by Zanitti et al. [24], diclofenac exposure was found to cause developmental imbalance in ovarian growth and trigger compensatory activation of the antioxidant defense system. The observation of diclofenac-induced developmental interference across species, independent of the biological model, strengthens the hypothesis that it is closely linked to altered redox homeostasis and endocrine disruption.

The significant sensitivity detected on day 7 may be related, at least in part, to stage-specific differences in drug uptake. However, such mechanisms at the carrier level were not directly measured in this study and are discussed here as possible hypotheses derived from the previous literature. Vujica et al. [25] identified the *Oatp1d1* (Organic Anion Transporter Polypeptide 1d1) membrane transporter as a crucial mediator of diclofenac toxicity and demonstrated that functionally inactivating this transporter significantly reduced toxic outcomes. In this context, increased activity of these transporters in early-stage embryos facilitates the accumulation of diclofenac in developing tissues, leading to the severe embryotoxic effects observed during organogenesis.

At the cellular level, quantitative proteomic analyses conducted by Sun et al. [26] demonstrated that diclofenac disrupts purine metabolism, triggering mitochondrial dysfunction and apoptosis as a result.

Early embryonic development is a critical period for cardiovascular morphogenesis. Chen, Gao, Zhang, Zhang, Zhou, Li and Gao [21] demonstrated that diclofenac disrupts heart development by suppressing the Wnt signaling pathway, particularly wnt3a and gata4, leading to serious functional and structural abnormalities such as pericardial edema. In a study, Ghosh et al. [27] demonstrated that diclofenac induces proteasome inhibition in cardiomyocytes, leading to mitochondrial dysfunction and subsequent cell death in cardiac tissues. The fundamental role of the Wnt signaling pathway in maintaining embryonic tissue homeostasis and regulating organogenesis has been more prominently emphasized in recently published high-impact studies. It has been reported that disruptions in this signaling pathway cause irreversible damage not only to heart development but also to nervous and skeletal morphogenesis [28]. Consequently, it may be hypothesized that the high mortality rate of 93.33% observed in the 7-day group reflects a combination of mechanisms reported in other models, such as Oatp1d1-mediated diclofenac uptake, secondary mitochondrial dysfunction, abnormalities in purine metabolism, and Wnt pathway-mediated organogenesis interactions. Since none of these mechanisms were directly tested in the present study, they are proposed only as potential explanations that could be validated by specific mechanistic experiments.

Additionally, transcription abnormalities in circadian rhythm genes (bmal1) observed in zebrafish larvae exposed to diclofenac by Sulukan [13] provide important insights into the molecular and epigenetic mechanisms underlying the growth retardation observed in our study. Similar findings in mammalian models emphasize the persistence of these early-stage damages. Krebs Ribeiro et al. [29] reported that prenatal diclofenac exposure in rats not only delayed pubertal development but also disrupted adult sexual behavior patterns. Although diclofenac applied to zebrafish embryos is a weak genotoxicant, Martins et al. [30] demonstrated that it is responsible for significant acute toxicopathological effects such as mortality and developmental malformations. This suggests that the developmental arrest observed in our in ovo model may lead to long-term physiological consequences. The results obtained on Day 7 in the study are also supported by stereological evidence showing that prenatal exposure altered the morphology of the cervical spinal cord and significantly affected neuronal numerical density [31].

In contrast, the increased resistance of embryos to the drug on day 14 (indicated by the rise in LD50) can be attributed to the maturation of metabolic capacity. This increased resistance on day 14 is in agreement with the statistically determined 55% increase in the LD50 value (rising from 20.67 mg/kg to 32.16 mg/kg). This finding is consistent with the significant rise in hepatic cytochrome P450 activities and the maturation of xenobiotic metabolism capacity previously reported to occur in chicken embryos during the latter half of incubation (post-day 14). Furthermore, DNA adduct formation models have demonstrated that the capacity of the chicken fetal liver to metabolize xenobiotics and repair DNA damage is substantially enhanced during advanced developmental stages [32]. Hamilton et al. [33] revealed that hepatic aryl hydrocarbon hydroxylase (AHH) activity and the microsomal mixed-function oxidase (MFO) system undergo extensive functional development during this period, thereby providing the late-stage embryo with a robust mechanism for xenobiotic detoxification.

In future studies, the use of transcriptomic analyses (RNA-seq), as applied by Hernández-Zamora et al. [34] in aquatic organisms, to uncover the genetic background of this toxic profile will be useful for revealing the mechanisms in greater detail. Furthermore, supporting the findings with organoid models that are closer to human physiology will take risk assessment to a more advanced level [35].

This study demonstrates that the embryolethal effect of diclofenac is dependent on dose and stage of administration. This study quantitatively demonstrates that the embryolethal effect of diclofenac sodium shows a statistically significant interaction not only with dose but also with timing of exposure (via LD50 and Potency Ratio). The high sensitivity observed in chicken embryos on day 7 is consistent with the concept of stage-dependent sensitivity during early organogenesis, whereas the increased resistance on day 14 is consistent with the maturation of xenobiotic metabolism in avian embryos, as previously reported. Within the limitations of this avian in ovo model, these findings provide model-specific preliminary data for the comparative developmental toxicology of NSAIDs and a One Health-focused assessment of drug exposure across animal species. They should not be interpreted as direct evidence of risk during human pregnancy or as a basis for changes in clinical prescribing practices. Limitations. Certain limitations of this study should be noted. First, only a single species and developmental model (chicken embryo, in ovo) were used. This model does not reflect maternal metabolism, placental transfer, mammalian pharmacokinetics, or the full complexity of mammalian pregnancy. Therefore, the findings cannot be directly extrapolated to humans or veterinary species other than birds. Second, a proprietary commercial diclofenac formulation (excipients: sodium metabisulfite (E223), propylene glycol, benzyl alcohol (E1519), N-methyl-2-pyrrolidone, sodium hydroxide, water for injection) was used instead of a pure analytical standard. Although saline vehicle control was included, effects attributable to excipients cannot be entirely ruled out. Third, only two embryonic stages (day 7 and day 14) were examined; intermediate stages, which may exhibit different sensitivity, were not investigated. Fourth, the primary endpoint of the study was limited to mortality (embryo mortality rate); detailed analyses such as gene/carrier expression, CYP450 activity, and oxidative stress, as well as morphological, biochemical, and histopathological parameters, were excluded from the evaluation. Therefore, mechanistic assessments regarding CYP450, Oatp1d1, the Wnt signaling pathway, mitochondrial dysfunction, or purine metabolism are presented not as direct findings of this study, but as reasonable hypotheses derived from literature data. Fifth, although the width of the 95% confidence interval for the 7-day LD50 estimate indicates low precision of the point estimate, the inter-stage comparisons at matching doses retain their statistical validity. Finally, the in ovo model, which is based on direct embryonic exposure, is best considered a screening tool; the data obtained from this model must be validated in mammalian systems and clinical studies.

## 5. Conclusions

The study found that early-stage exposure to diclofenac is significantly more toxic than late-stage exposure. While these data provide valuable insights regarding animal health and development, they should not be interpreted as a direct indicator of risk for human pregnancy. To fully elucidate the mechanisms and conduct interspecies risk assessments, more comprehensive research incorporating biochemical, molecular, and mammalian models is needed.

## Figures and Tables

**Table 1:** Mortality rates of chicken embryos on days 7 and 14 following in ovo administration of various doses of diclofenac.

Dose (mg/kg)	Day	N	EED/LED	Death Rate (%)	Actual Death Rate (Abbott %)	P1 (Dose)	P2 (Stage)
Control	7	30	0/0	0.0	-	-	-
	14	30	0/1	3.3	-	-	>0.05
Saline	7	30	0/0	0.0	-	>0.05	-
	14	30	0/1	3.3	-	>0.05	>0.05
3.125	7	30	0/0	0.0	0.0	>0.05	-
	14	30	0/1	3.3	0.0	>0.05	>0.05
6.25	7	30	0/0	0.0	0.0	>0.05	-
	14	30	0/1	3.3	0.0	>0.05	>0.05
12.5	7	30	10/0	33.3	33.3	<0.01	<0.001 *
	14	30	0/0	0.0	0.0	>0.05	
25	7	30	25/1	86.7	86.7	<0.001	<0.001 *
	14	30	0/12	40.0	37.9	<0.01	
50	7	30	28/0	93.3	93.3	<0.001	>0.05
	14	30	0/26	86.7	86.2	<0.001	

N: Total number of fertile eggs per group; EED: Early embryonic death (mortality occurring between days 0–7 of incubation); LED: Late embryonic death (mortality occurring between days 8–21 of incubation); Actual Death Rate (Abbott): A formula used to calculate the actual death rate by correcting for natural mortality in the control group; negative values, which arise mathematically when treatment-group mortality is below control mortality and have no biological meaning, were set to 0.0%. P1: Statistical significance of the dose-dependent increase in mortality compared to the respective control group (Pearson Chi-square; Holm–Bonferroni-adjusted); P2: Statistical significance of the difference in mortality between Day 7 and Day 14 for the same dose (Fisher’s Exact Test). * *p* < 0.05 indicates statistical significance.

## Data Availability

The original contributions presented in this study are included in the article material. Further inquiries can be directed to the corresponding author.

## Abbreviations

The following abbreviations are used in this manuscript:

NSAIDs	Nonsteroidal anti-inflammatory drugs
COX-1	Cyclooxygenase-1
COX-2	Cyclooxygenase-2
FDA	Food and Drug Administration
CKD	Chronic kidney disease
LD50	Lethal Dose 50
NAE	Number of normal alive embryos
EED	Early embryonic death
LED	Late embryonic death
Oatp1d1	Organic Anion Transporter Polypeptide 1d1
